# Characteristics and Outcomes of Children With Severe Acute Respiratory Syndrome Coronavirus 2 (SARS-CoV-2) Infection Admitted to a Quaternary Hospital: A Single-Center Experience

**DOI:** 10.7759/cureus.52532

**Published:** 2024-01-18

**Authors:** Saleh S Alshehri, Bushra I Minhaji, Mohsina R Pasha, Dina Fouda, Jency Joseph, Nehad Ahmed

**Affiliations:** 1 Pediatric Intensive Care Unit, King Saud Medical City, Riyadh, SAU; 2 Pharmaceutical Care Services, King Saud Medical City, Riyadh, SAU; 3 Nursing Administration, King Saud Medical City, Riyadh, SAU; 4 Clinical Pharmacy, Prince Sattam bin Abdulaziz University, Alkharj, SAU

**Keywords:** multisystem inflammatory syndrome in children (mis-c), coronary artery aneurysm, continuous renal replacement therapy (crrt), pims-ts, steroids, covid-19 in children, pediatric covid-19, pediatric icu, sars-cov-2, pediatric coronavirus 2019 complications

## Abstract

Objectives

In the setting of the recent global pandemic, children infected with the severe acute respiratory syndrome coronavirus 2 (SARS-CoV-2) virus causing the coronavirus disease 2019 (COVID-19) presented to our hospital with a variety of symptoms ranging from mild to severe disease including multiorgan dysfunction. Our objective was to study the clinical profile, risk factors, complications, and outcomes in pediatric patients admitted to our center with SARS-CoV-2 infection.

Methods

This retrospective observational study was conducted at a large quaternary center in Riyadh between May 2020 and September 2021. The study population was comprised of children between 0 and ≤14 years with SARS-CoV-2 suspicion or positivity.

Results

One hundred and fifty-six children were included in the study, the majority of whom were 1-10 years old. One hundred and twenty of them (76.93%) were SARS-CoV-2 positive. Fifty-nine patients (37.18%) were labelled as multisystem inflammatory syndrome in children (MIS-C) based on clinical and lab criteria, of whom 35 (22.44%) tested SARS-CoV-2 positive. Hematological disease was found to be the most common comorbidity, followed by neurological and chronic lung diseases. The most common symptoms encountered were fever, cough, vomiting, fatigue, and diarrhea. Eighty patients (51%) required pediatric intensive care unit (PICU) admission (length of stay: 5-12 days), among whom 32 (40%) required ventilation, 26 (32.5%) needed hemodynamic support, and three patients (3.75%) underwent continuous renal replacement therapy (CRRT). The overall mortality rate was 4.5% (seven patients) among the studied population. The most frequent lab abnormalities were found to be elevated serum ferritin, erythrocyte sedimentation rate (ESR), C-reactive protein (CRP), and lactate dehydrogenase (LDH) levels. Ninety-one percent received antibiotics, and prophylactic anticoagulant was used in 32%. In the MIS-C subset, 80.5% received steroids, 71.43% intravenous immunoglobulin (IVIG), and 5.17% (three patients) tocilizumab.

Conclusion

The SARS-CoV-2 infection presented with a range of severity among our cohort of children; however, most of the patients responded well to appropriate supportive treatment. A slight male preponderance was noted. The most common symptoms encountered were fever, cough, vomiting, fatigue, and diarrhea. Inflammatory markers such as ESR, CRP, serum ferritin, and LDH levels were found to be elevated in nearly all patients. Raised serum lactate and serum creatinine and lymphopenia were of significant note in patients with MIS-C. Higher mortality rates were observed in patients with MIS-C and those requiring respiratory support. In addition to these two factors, the presence of comorbidities and the need for CRRT were associated with prolonged PICU length of stay.

## Introduction

The coronavirus disease 2019 (COVID-19) outbreak started in Wuhan, China, in November 2019 as pneumonia of unknown cause and rapidly spread to the rest of the world. On January 30, 2020, the World Health Organization (WHO) declared COVID-19 a global health emergency; by March 11, 2020, it was acknowledged as a global pandemic. Children constituted only around 1-2% of total cases [1] with milder presentation initially [2] and later involved neonates to adolescents, ranging from asymptomatic infection to critical illness with multiorgan failure. The highest proportion was seen in children over 10 years in a European study by Götzinger et al. [3]; hospitalization rate was also significantly higher in Europe (60%) as compared to 5.7-20% in the USA [4,5].

Although the incidence of pediatric severe acute respiratory syndrome coronavirus 2 (SARS-CoV-2) infection was originally reported to be between 0.8% and 2.0% of all cases in Europe and the USA [5-7], this figure kept evolving with time, and Saudi Arabian studies reported up to 4% incidence [8,9]. Children having comorbidities were found to have higher pediatric intensive care unit (PICU) admission rates and risk of mortality.

A distinct clinical entity soon emerged in children associated with systemic hyperinflammatory response, termed as multisystem inflammatory syndrome in children (MIS-C), also known as pediatric inflammatory multisystem syndrome temporally associated with SARS-CoV-2 (PIMS-Ts). It was first reported in Europe and then worldwide [4,10,11], with varying severities and multisystem involvement including cardiac and gastrointestinal (GI) symptoms, skin rash, conjunctivitis, and shock. MIS-C has some overlapping features with Kawasaki syndrome but occurs in higher age group, previously healthy school-aged children, and recovery is usually rapid and complete without sequelae [3].

Plethoric data is available regarding the disease manifestation in both adults and children at this point, but this study presents our experience in the pediatric age group admitted with SARS-CoV-2 infection and those presenting with clinical manifestations of MIS-C at a large quaternary referral center in Saudi Arabia.

This article was previously presented in summary as a poster presentation at the 2022 Saudi Critical Care Society (SCCS) Annual International Critical Care Conference in Jeddah, Saudi Arabia, on October 4, 2022.

## Materials and methods

Permission to conduct the study was granted by the Institutional Review Board (IRB) of King Saud Medical City in Riyadh, Saudi Arabia (approval number: H1RI-09-Aug20-03). The study included pediatric patients aged between 0 and ≤14 years, who were admitted to a single quaternary hospital (King Saud Medical City) between May 2020 and September 2021. A standardized electronic medical record and patients' medical files were used to gather patient demographic, clinical, and laboratory data. Children who tested positive for SARS-CoV-2 or those suspected to have SARS-CoV-2 infection were included in the study. As the study was retrospective with no individual identifying data, the requirement for informed patient consent was waived. All children presenting to the pediatric ER during a period of 16 months, from May 2020 to September 2021, ranging from neonates up to 14 years of age and having fever, with or without respiratory symptoms, gastrointestinal (GI) symptoms, and hemodynamic instability, were included in the study as well as asymptomatic children with a history of exposure to a confirmed case of SARS-CoV-2. A total of 156 patients were included in the final data collection. Patients were admitted in both the general ward and PICU. Children above 14 years and those with incomplete data were excluded from the study.

Children having confirmed SARS-CoV-2 infection (by positive polymerase chain reaction (PCR) nasopharyngeal swab test) and those with strong clinical suspicion of COVID-19 or MIS-C were included in data collection. Data was collected for patients' demographic characteristics, presenting symptoms and signs, associated comorbidities, laboratory data, need for PICU admission, level of respiratory and hemodynamic support required, and treatment. Patients from the ER were identified through the COVID-19 Score, designed by the Ministry of Health of Saudi Arabia, which included a history of contact with an individual with SARS-CoV-2 infection or recent travel abroad, along with defined presenting symptoms such as fever, cough, shortness of breath, nausea, vomiting, and diarrhea. The lab results were collected for hematological parameters such as leukopenia, leukocytosis, neutropenia or neutrophilia, thrombocytopenia, deranged coagulation profile, and acute-phase reactants. Mortality rate, length of stay (LOS), PICU admission, and need for mechanical ventilation, inotropic support, or continuous renal replacement therapy (CRRT) were reported as indicators of clinical outcome.

The patients were categorized as those (1) positive for SARS-CoV-2 and had MIS-C; (2) negative for SARS-CoV-2 but had MIS-C; (3) positive for SARS-CoV-2 but did not have MIS-C; and (4) negative for SARS-CoV-2 as well as MIS-C. The children were classified as neonate (0-30 days), infants (1 month-≤1 year), child (1-≤10 years), and adolescent (10-≤14 years). Unless specified according to age, all have been generally referred to as "child" for ease of reference.

The collected data included patients' demographic and clinical characteristics, presenting symptoms of children admitted with a confirmed or suspected diagnosis of SARS-CoV-2, the medications used for management, laboratory criteria on admission, and the outcomes of treatment. The data was collected and analyzed descriptively using Microsoft Excel (Microsoft Corporation, Redmond, Washington, United States) and the chi-squared test. Results were represented as numbers and ratios, and p-values were calculated to denote significance.

## Results

During the study period, 156 patients were admitted to the pediatric department. More than 54% of the children did not have MIS-C but tested positive for SARS-CoV-2 (85/156 patients), and 22.44% of them had MIS-C and also tested positive for SARS-CoV-2 (35/156 patients).

About 51.92% of the patients were children (81/156 patients) and 26.92% of them were infants (42/156 patients). More than half of the patients were males (82/156 patients). Only 43.59% of the patients had confirmed contact with SARS-CoV-2 patients (68/156 patients). About 57.69% of the patients had at least one comorbidity (90/156 patients). The most frequent comorbidities were hematological disease (13.46%, 21/156 patients), neurological disease (10.26%, 16/156 patients), chronic lung disease (8.97%, 14/156 patients), heart disease (8.33%, 13/156 patients), GI disease (7.69%, 12/156 patients), and metabolic/genetic disease (7.69%, 12/156 patients) (Table 1).

**Table 1:** The demographic characteristics of children admitted with a confirmed or suspected diagnosis of SARS-CoV-2 infection SARS-CoV-2: severe acute respiratory syndrome coronavirus 2

Characteristics	Number	Percentage (%)
Age group		
Neonates	11	7.05
Infants	42	26.92
Children	81	51.92
Adolescents	22	14.10
Sex		
Female	74	47.44
Male	82	52.56
Nationality		
Non-Saudi	61	39.10
Saudi	95	60.90
Exposure to SARS-CoV-2-infected individual		
Yes	68	43.59
Unknown	88	56.41
Comorbidities		
Chronic lung disease	14	8.97
Neurological disease	16	10.26
Hematological disease	21	13.46
Heart disease	13	8.33
Immune disease	2	1.28
Diabetes	1	0.64
Malignancy	7	4.49
Kidney disease	8	5.13
Gastrointestinal disease	12	7.69
Metabolic/genetic disease	12	7.69
Preterm	8	5.13

The median duration of symptoms before presentation was 3.6 days. Only 8.40% of the patients who were positive or had a history of exposure showed no symptoms (11 out of 131 patients). The most frequent symptoms for the patients were fever, vomiting, cough, fatigue, diarrhea, and shortness of breath (Table 2).

**Table 2:** The presenting symptoms of children admitted with a confirmed or suspected diagnosis of SARS-CoV-2 infection MIS-C: multisystem inflammatory syndrome in children; SARS-CoV-2: severe acute respiratory syndrome coronavirus 2; SOB: shortness of breath

Symptoms	Confirmed MIS-C (positive SARS-CoV-2 infection) (N=35)	Confirmed MIS-C (negative SARS-CoV-2 infection) (N=23)	No MIS-C with positive SARS-CoV-2 infection (N=85)	No MIS-C with negative SARS-CoV-2 infection (N=13)
Fever	30 (85.71%)	21 (91.30%)	55 (64.71%)	10 (76.92%)
Cough	18 (51.43%)	11 (47.83%)	29 (34.12%)	5 (38.46%)
SOB	14 (40%)	10 (43.48%)	20 (23.53%)	4 (30.77%)
Sore throat	3 (8.57%)	3 (13.04%)	0 (0%)	0 (0%)
Rhinorrhea	6 (17.14%)	2 (8.70%)	11 (12.94%)	1 (7.69%)
Cyanosis	7 (20%)	5 (21.74%)	6 (7.06%)	1 (7.69%)
Apnea	5 (14.29%)	0 (0%)	2 (2.35%)	0 (0%)
Respiratory distress	13 (37.14%)	12 (52.17%)	12 (14.12%)	2 (15.38%)
Crackles	9 (25.71%)	5 (21.74%)	8 (9.41%)	2 (15.38%)
Wheeze	4 (11.43%)	4 (17.39%)	8 (9.41%)	2 (15.38%)
Headache	3 (8.57%)	2 (8.70%)	1 (1.18%)	1 (7.69%)
Poor feeding	13 (37.14%)	11 (47.83%)	19 (22.35%)	2 (15.38%)
Abdominal pain	9 (25.71%)	6 (26.09%)	14 (16.47%)	2 (15.38%)
Nausea	5 (14.29%)	4 (17.39%)	2 (2.35%)	0 (0%)
Vomiting	19 (54.29%)	13 (56.52%)	23 (27.06%)	5 (38.46%)
Diarrhea	15 (42.86%)	13 (56.52%)	21 (24.71%)	4 (30.77%)
Decrease activity/fatigue	17 (48.57%)	13 (56.52%)	21 (24.71%)	5 (38.46%)
Dehydration	10 (28.57%)	7 (30.43%)	0 (0%)	4 (30.77%)
Shock	6 (17.14%)	12 (52.17%)	3 (3.53%)	2 (15.38%)
Rash	5 (14.29%)	6 (26.09%)	1 (1.18%)	1 (7.69%)
Conjunctivitis	1 (2.86%)	2 (8.70%)	0 (0%)	0 (0%)
Cracked lips	1 (2.86%)	1 (4.35%)	1 (1.18%)	0 (0%)
Extremity edema	4 (11.43%)	3 (13.04%)	2 (2.35%)	0 (0%)
Irritability	1 (2.86%)	5 (21.74%)	5 (5.88%)	0 (0%)
Loss of smell and taste	0 (0%)	0 (0%)	0 (0%)	0 (0%)
Lymphadenopathy	1 (2.86%)	1 (4.35%)	2 (2.35%)	1 (7.69%)
Myalgia	3 (8.57%)	1 (4.35%)	5 (5.88%)	0 (0%)
Seizures	5 (14.29%)	4 (17.39%)	10 (11.76%)	2 (15.38%)

The medications administered to the cohort are described in the following table (Table 3). The most prescribed medications were empirical antibiotics (91.03%, 142/156 patients) followed by steroids (45.51%, 71/156 patients).

**Table 3:** The management of children admitted with a confirmed or suspected diagnosis of SARS-CoV-2 MIS-C: multisystem inflammatory syndrome in children; SARS-CoV-2: severe acute respiratory syndrome coronavirus 2; HCQ: hydroxychloroquine; IVIG: intravenous immunoglobulin

Medications	MIS-C with positive SARS-CoV-2 infection (N=35)	MIS-C with negative SARS-CoV-2 infection (N=23)	No MIS-C, positive SARS-CoV-2 infection (N=85)	No MIS-C, negative SARS-CoV-2 infection (N=13)
Sedation	11 (31.43%)	11 (47.83%)	6 (7.06%)	4 (30.77%)
Midazolam	9 (25.71%)	10 (43.48%)	2 (2.35%)	3 (23.08%)
Fentanyl	7 (19.20%)	9 (39.13%)	2 (2.35%)	2 (15.38%)
Morphine	2 (5.71%)	2 (8.70%)	3 (3.53%)	0 (0%)
Inotropic support	11 (31.43%)	14 (60.87%)	1 (1.18%)	0 (0%)
Epinephrine	8 (22.86%)	8 (34.78%)	1 (1.18%)	0 (0%)
Norepinephrine	4 (11.43%)	6 (26.09%)	0 (0%)	0 (0%)
Dobutamine	1 (2.86%)	2 (8.70%)	0 (0%)	0 (0%)
Milrinone	3 (8.57%)	5 (21.74%)	0 (0%)	0 (0%)
Empirical antibiotics	33 (94.29%)	23 (100%)	73 (85.88%)	13 (100%)
Steroids	29 (82.86%)	18 (78.26%)	17 (20%)	7 (53.85%)
Dexamethasone	9 (25.71%)	3 (13.04%)	4 (4.71%)	2 (15.38%)
Methylprednisolone	20 (57.14%)	14 (60.87%)	7 (8.24%)	6 (46.15%)
Hydrocortisone	1 (2.86%)	1 (4.35%)	2 (2.35%)	0 (0%)
Prednisolone	3 (8.57%)	1 (4.35%)	7 (8.24%)	1 (7.69%)
Enoxaparin	21 (60%)	17 (73.91%)	11 (12.94%)	1 (7.69%)
Aspirin	4 (11.43%)	5 (21.74%)	0 (0%)	0 (0%)
Antiviral	3 (8.57%)	7 (30.43%)	0 (0%)	1 (7.69%)
HCQ	3 (8.57%)	0 (0%)	7 (8.24%)	1 (7.69%)
Tocilizumab	1 (2.86%)	2 (8.70%)	0 (0%)	0 (0%)
IVIG	17 (48.57%)	15 (65.22%)	4 (4.71%)	0 (0%)

Lab analysis

One hundred and twenty patients (76.9%) tested positive for SARS-CoV-2 reverse transcription (RT)-PCR, while 36 (23%) tested negative. COVID-19 serology was not done in our patients. Lymphopenia was observed in patients with MIS-C. Platelet count was essentially normal among the study participants. Mean hemoglobin (Hb) was low. Blood urea nitrogen (BUN) and serum creatinine were raised among patients with MIS-C while being normal in children without MIS-C. Average D-dimer was increased in all subgroups of patients. Elevated transaminases were seen in patients with MIS-C, especially aspartate transaminase (AST). Blood gases were maintained within normal range, although patients with MIS-C demonstrated higher lactate levels. Inflammatory markers such as ferritin, erythrocyte sedimentation rate (ESR), C-reactive protein (CRP), and lactate dehydrogenase (LDH) were found to be elevated in almost all patients along with a slight increase in D-dimer levels and white blood cell (WBC) count and a low average Hb level. Of note, serum creatinine and lactate were significantly higher in those suspected or confirmed to have MIS-C (Table 4).

**Table 4:** Laboratory criteria on admission for children admitted with a confirmed or suspected diagnosis of SARS-CoV-2 MIS-C: multisystem inflammatory syndrome in children; SARS-CoV-2: severe acute respiratory syndrome coronavirus 2; LDH: lactate dehydrogenase; CRP: C-reactive protein; ESR: erythrocyte sedimentation rate; PT: prothrombin time; PTT: partial thromboplastin time; INR: international normalised ratio; Hgb: hemoglobin; WBC: white blood cell; BUN: blood urea nitrogen; AST: aspartate transaminase; ALT: alanine transaminase

Laboratory tests (average)	Normal range	MIS-C and positive SARS-CoV-2 infection (N=35)	MIS-C and negative SARS-CoV-2 infection (N=23)	No MIS-C and positive SARS-CoV-2 infection (N=85)	No MIS-C and negative SARS-CoV-2 infection (N=13)
Ferritin (µg/L)	Less than 336	980.78	824.63	468.79	389.35
D-dimer	Less than 0.50	4.24	4.42	4.23	2.35
Fibrinogen (g/L)	2-4	4.02	3.79	3.78	2.87
LDH (IU/L)	105-333	1143.91	709.94	488	735.50
CRP (mg/L)	Less than 10	77.35	80.82	69.99	38.91
ESR (mm/hr)	1-13 (males) and 1-20 (females)	41.44	52.85	45.96	45.86
PT (seconds)	11-13.5	15.43	14.90	13.49	14.16
PTT (seconds)	25-35	37.85	38.29	34.68	35.98
INR	0.8-1.1	1.09	1.18	1.11	1.17
Platelets (×10^9^/L)	150-450	267.84	255.91	341.26	342.09
Hgb (g/dL)	11.5-16	10.49	10.32	10.51	11.56
WBC count (×10^9^/L)	4.5-11.0	11.39	13.04	12.03	11.34
Neutrophils	40-60%	63.77	65.64	47.73	47.65
Lymphocytes	20-40%	26.65	25.88	40.96	37.54
BUN (mmol/L)	2-6	8.82	8	3.88	4.57
Creatinine (micromoles/L)	20-65	99.85	89	36.07	29.8
Albumin (g/L)	35-50	35.49	32.87	39.05	37.18
AST (U/L)	5-40	230.17	76.80	36.81	44.57
ALT (U/L)	7-55	140.37	38.26	20.74	40.43
Lactate (mmol/L)	0.5-2.2	2.36	3.48	1.68	2.13

The mortality rate was 4.5% (among the 156 patients, seven patients died), all of whom were confirmed to have MIS-C. Most of the patients who had MIS-C were admitted to the PICU.

More than 97% of the patients who had confirmed MIS-C and had a positive SARS-CoV-2 test were admitted to PICU for an average of 7.05 days (34/35 patients). The average hospital LOS was 11.69 days, and most of the patients did not require any respiratory support (58.97%, 92/156 patients). About 17% of the patients required invasive mechanical ventilation (26/156 patients), and 14.74% of the patients were on low-flow oxygen (23/156 patients). About 48% of the patients were on the ventilator for less than five days (75/156 patients), 37.93% for 5-10 days (59/156 patients), and 13.79% for more than 10 days (22/156 patients). Only 1.92% (three patients) required CRRT support (Table 5).

**Table 5:** Outcomes of children admitted with a confirmed or suspected diagnosis of SARS-CoV-2 MIS-C: multisystem inflammatory syndrome in children; SARS-CoV-2: severe acute respiratory syndrome coronavirus 2; PICU: pediatric intensive care unit; LOS: length of stay; DNR: do not resuscitate; HFOV: high-frequency oscillatory ventilation; MV: mechanical ventilation; HFNC: high-flow nasal cannula; NC: nasal cannula; CRRT: continuous renal replacement therapy

Outcomes	MIS-C with positive SARS-CoV-2 infection (N=35)	MIS-C with negative SARS-CoV-2 infection (N=23)	No MIS-C with positive SARS-CoV-2 infection (N=85)	No MIS-C with negative SARS-CoV-2 infection (N=13)
Outcome, no. (%)				
Died	5 (14.29%)	2 (8.6%)	0 (0%)	0 (0%)
Survived	30 (85.71%)	21 (91.3%)	85 (100%)	13 (100%)
PICU admission, no. (%)	34 (97.14%)	21 (91.30%)	19 (22.35%)	6 (46.15%)
Average PICU LOS in days	7.05	11.79	5.61	12.33
Average hospital LOS in days	12.97	15.09	10.14	12.38
DNR, no. (%)	1 (2.86%)	0 (0%)	0 (0%)	1 (7.69%)
Respiratory support, no. (%)	22 (62.86%)	17 (73.91%)	19 (22.35%)	6 (46.15%)
Level of respiratory support, no. (%)				
HFOV	2 (5.71%)	3 (13.04%)	1 (1.17%)	0 (0%)
Invasive MV	9 (25.71%)	9 (39.13%)	4 (4.71%)	4 (30.77%)
Non-Invasive MV	3 (8.57%)	2 (8.70%)	3 (3.53%)	0 (0%)
HFNC	1 (2.86%)	0 (0%)	0 (0%)	0 (0%)
Low-flow oxygen (NC)	7 (20%)	3 (13.04%)	11 (12.94%)	2 (15.38%)
Room air	13 (37.15%)	6 (26.09%)	66 (77.65%)	7 (53.85%)
CRRT support, no. (%)	2 (5.71%)	1 (4.35%)	0 (0%)	0 (0%)

Factors associated with increased in-hospital mortality were noticed to be a requirement of respiratory support and those who had MIS-C (Table 6). The same factors were also found in those who needed PICU admission, in addition to coexisting illnesses and CRRT requirements (Table 7). Together, all four of these factors were the major contributors to prolonged LOS in PICU (Table 8).

**Table 6:** Factors associated with in-hospital mortality MIS-C: multisystem inflammatory syndrome in children; CRRT: continuous renal replacement therapy

Variables	Survived	Died	P-value
Age group			
Neonates	90.91% (10/11)	9.09% (1/11)	0.496
Infants	92.86% (39/42)	7.14% (3/42)	
Children	96.30% (78/81)	3.70% (3/81)	
Adolescents	100% (22/22)	0% (0/22)	
Gender			
Male	97.56% (80/82)	2.44% (2/82)	0.193
Female	93.24% (69/74)	6.76% (5/74)	
Criteria fulfilling MIS-C			
Yes	87.93% (51/58)	12.06% (7/58)	0.039
No	100% (98/98)	0% (0/98)	
Coexisting illnesses			
Yes	93.33% (84/90)	6.67% (6/90)	0.125
No	98.48% (65/66)	1.52% (1/66)	
Respiratory support			
Yes	89.06% (57/64)	10.94% (7/64)	0.001
No	100% (92/92)	0% (0/92)	
CRRT support			
Yes	66.66% (2/3)	33.33% (1/3)	0.089
No	96.07% (147/153)	3.92% (6/153)	

**Table 7:** Factors associated with PICU admission MIS-C: multisystem inflammatory syndrome in children; CRRT: continuous renal replacement therapy: PICU: pediatric intensive care unit

Variables	Admitted to PICU	Not admitted	P-value
Age group			
Neonates	27.27% (3/11)	72.73% (8/11)	0.104
Infants	45.24% (19/42)	54.76% (23/42)	
Children	62.96% (51/81)	37.04% (30/81)	
Adolescents	31.82% (7/22)	68.18% (15/22)	
Gender			
Male	48.78% (40/82)	51.21% (42/82)	0.542
Female	54.05% (40/74)	45.94% (34/74)	
Criteria fulfilling MIS-C			
Yes	91.37% (53/58)	8.62% (5/58)	0.0001
No	27.55% (27/98)	72.44% (71/98)	
Coexisting illnesses			
Yes	54.44% (49/90)	45.56% (41/90)	0.041
No	37.88% (25/66)	62.12% (41/66)	
Respiratory support			
Yes	85.94% (55/64)	14.06% (9/64)	0.0001
No	20.65% (19/92)	79.35% (73/92)	
CRRT support			
Yes	100% (3/3)	0% (0/3)	0.017
No	50.32% (77/153)	49.67% (76/153)	

**Table 8:** Factors associated with prolonged length of stay in PICU MIS-C: multisystem inflammatory syndrome in children; CRRT: continuous renal replacement therapy; PICU: pediatric intensive care unit

Variables in days	Less than 10	10-19	20-29	30-39	40-49	More than 49	P-value
Age group							
Neonates	81.82% (9/11)	18.18% (2/11)	0% (0/11)	0% (0/11)	0% (0/11)	0% (0/11)	0.987
Infants	61.90% (26/42)	21.43% (9/42)	4.76% (2/42)	4.76% (2/42)	2.38% (1/42)	4.76% (2/42)	
Children	64.20% (52/81)	20.99% (17/81)	3.70% (3/81)	3.70% (3/81)	4.94% (4/81)	2.47% (2/81)	
Adolescents	68.18% (15/22)	22.73% (5/22)	4.54% (1/22)	0% (0/22)	0% (0/22)	4.54% (1/22)	
Gender							
Male	63.41% (52/82)	21.95% (18/82)	2.44% (2/82)	3.66% (3/82)	4.88% (4/82)	3.66% (3/82)	0.735
Female	67.57% (50/74)	20.27% (15/74)	5.40% (4/74)	2.70% (2/74)	1.35% (1/74)	2.70% (2/74)	
Criteria fulfilling MIS-C							
Yes	39.66% (23/58)	36.20% (21/58)	10.34% (6/58)	3.45% (2/58)	6.90% (4/58)	3.45% (2/58)	0.001
No	73.47% (72/98)	16.33% (16/98)	3.06% (3/98)	3.06% (3/98)	1.02% (1/98)	3.06% (3/98)	
Coexisting illnesses							
Yes	54.44% (49/90)	27.78% (25/90)	5.56% (5/90)	3.33% (3/90)	3.33% (3/90)	5.56% (5/90)	0.018
No	80.30% (53/66)	12.12% (8/66)	1.52% (1/66)	3.03% (2/66)	3.03% (2/66)	0% (0/66)	
Respiratory support							
Yes	43.75% (28/64)	32.81% (21/64)	4.69% (3/64)	6.25% (4/64)	6.25% (4/64)	6.25% (4/64)	0.0001
No	80.43% (74/92)	13.04% (12/92)	3.26% (3/92)	1.09% (1/92)	1.09% (1/92)	1.09% (1/92)	
CRRT support							
Yes	33.33% (1/3)	0% (0/3)	0% (0/3)	33.33% (1/3)	0% (0/3)	33.33% (1/3)	0.0001
No	66.01% (101/153)	21.56% (33/153)	3.92% (6/153)	2.61% (4/153)	3.26% (5/153)	2.61% (4/153)	

## Discussion

To the best of our knowledge, this is one of the largest studies on pediatric SARS-CoV-2 infection, from a single quaternary care center in Saudi Arabia.

Interpretation of studied factors

The clinical profile of children infected with SARS-CoV-2 in our center was highly variable, ranging from asymptomatic infection to severely affected patients with multiorgan failure requiring invasive management like hemodynamic support, mechanical ventilation, and CRRT in a small percentage. The study cohort included a total of 156 children, out of which 76.9% (120) tested positive for SARS-CoV-2 RT-PCR, while 23.1% (36) tested negative. The percentage of males was observed to be marginally higher at 52.56%.

In our study participants, the majority was formed by children between one and 10 years (51.92%; 81), followed by infants (26.92%; 42), then adolescents (14.10%; 22), and neonates (7.05%, 11). In contrast, a meta-analysis studying SARS-CoV-2 infection in children found more than 50% of subjects to be adolescents [12]. A multicentric trial from our region has observed the maximum number of the affected population to be infants (less than 1-year-old) and 6-11 years as the second most affected age group [13]. DeBiasi et al. [14] too have reported that they found an over-representation of children less than one year and more than 17 years in their study as each of these categories represented 32% (14/44) of all hospitalized patients. Others noted an increased risk of severe disease in infants and adolescents (10-14 years) [3,15].

In our study, around 11 children (8.4%) were asymptomatic and were screened subsequent to contact exposure in the family. A meta-analysis by Irfan et al. reported this figure to be around 13% [12]. An early Chinese report also reported significant proportion of asymptomatic infection in children, unlike adults, along with several other international literature [16]. The exact prevalence of asymptomatic cases is unknown due to varying rates of testing in this group.

Some infants and neonates were admitted during the early pandemic period with a history of contact with patients positive for SARS-CoV-2 for the purpose of isolation and clinical observation, but most of them remained asymptomatic.

Of our patients, only 43.59% of the children had contact with a confirmed case of SARS-CoV-2 infection. Irfan et al. [12] have reported contact exposure to be 64% and travel history in 13%. Increased prevalence of asymptomatic population which was not screened for SARS-CoV-2 in the latter part of the pandemic could explain this discrepancy in rate of exposure, as asymptomatic infection could be a potential source of infection spread.

At least one underlying comorbidity was reported in 56.79% of patients, of which the most frequent was hematological 13.46%, especially sickle cell anemia (SCA), followed by chronic neurological disease (10.26%) and chronic lung disease (8.97%). 43.21% of our patients had no comorbidity. Some studies have found coexisting illnesses in as low as 27% of pediatric patients, the most commonly reported ones being immunosuppression and lung disease [12]. In a cohort of patients exclusively with MIS-C from the USA, 48% of patients had a comorbidity, most commonly asthma and allergic rhinitis [4].

The presence of underlying chronic medical conditions plays a crucial role by affecting pediatric SARS-CoV-2 disease severity and outcome as suggested by various studies [14,15]. However, there is a possibility of higher infection exposure in this population due to more frequent hospital visits for their underlying condition. Another possibility is an incidental finding of SARS-CoV-2 during hospitalization for chronic conditions and higher rate of investigations. There is increased risk of critical illness in children with obesity, intellectual disabilities, and seizure disorder [17]. A meta-analysis by Tsankov et al. reported that the risk of severe SARS-CoV-2 infection was 5.1% in children with underlying comorbidities while being only 0.2% in children without comorbidities; similarly, a relative risk for mortality was also higher in children with comorbidities [17].

We found the median duration of symptoms for SARS-CoV-2-infected children before presentation was 3.6 days. In patients exclusively with MIS-C from a US study [4], the median duration of symptoms before presentation was reported as 4.5 days (3-6 days). From our region, initially, other studies had reported mild to moderate symptoms in majority of children and not requiring critical care [13].

The most frequent symptoms in patients with SARS-CoV-2 infection (those without the manifestation of MIS-C) were found to be fever, cough, vomiting, diarrhea, and fatigue, followed by poor feeding, conjunctivitis, and respiratory distress. Anosmia could not be assessed effectively as it was a pediatric study. Other studies also suggested that presentations were more or less similar in different age groups with a higher prevalence of abdominal symptoms in children above five years of age [12].

Of the total patients, 59 (37.8%) had clinical manifestations of MIS-C (as it was a quaternary care center receiving relatively sicker patients from the region). From an international study, the overall incidence of MIS-C was found 5.1 (95% CI, 4.5-5.8) persons per 1,000,000 person-months, indicating it to be a rare complication of SARS-CoV-2 infection; this study was done over a period of three months, before the introduction of vaccination [18]. However, another multicentric study from the USA revealed that during the winter of 2020-2021, for children aged 5-11 years, there was one MIS-C hospitalization for every COVID-19 hospitalization, suggesting that MIS-C may not be as rare of a COVID-19 sequela as generally perceived [19].

The most common presenting symptoms in our patients with MIS-C were fever, vomiting, diarrhea, fatigue, cough, and shock. Children with MIS-C reported a higher incidence of respiratory distress and shock, and all cases with rashes were almost exclusively present in children with MIS-C (11 out of 13 patients). In our patients, the finding of conjunctivitis was found solely in patients with MIS-C. A similar pattern is described in many international studies. In children with MIS-C from the USA, the most frequent symptoms reported were fever, abdominal pain, nausea, vomiting, and hypotension [4]. In our study, the conditions associated with children presenting with MIS-C were found to be respiratory distress (43%) and shock (13.7%).

We noted the prevalence of seizures in our patient cohort to be around 13% (21 patients), of whom 10.26% (16 patients) were known to have preexisting neurological disorder. Headache was less frequently observed with only 4.5% (seven patients) manifesting the symptom. Similar figure of central nervous system (CNS) involvement, around 12% is reported from other studies [4,12]. The prevalence of neurological comorbidities in these studies was not significant. Significantly higher proportion of neurological presentation in pediatric SARS-CoV-2 (around 66.67%) was reported from one study [20].

GI symptoms such as vomiting (55%, 32 patients), diarrhea (48%, 28 patients), abdominal pain (25%, 15 patients), and nausea (15%, nine patients) were more common in children with MIS-C. At the beginning of the pandemic, six patients presented to our center with symptoms suggestive of appendicitis, and all underwent surgery for the same; of them, four were found to have features of suppurative appendicitis, but the other two had no such signs. Among those who had true appendicitis, three out of the four were found to be SARS-CoV-2 positive, whereas in the two children having normal intraoperative finding, one was positive and another was negative for SARS-CoV-2.

We had an ICU admission rate of 51.2%, which was quite high compared to other studies. This is because threshold for PICU admission for acute symptomatic SARS-CoV-2 was high in our hospital and also because it serves as a quaternary care center in the region. Some patients were admitted only for clinical observation in the initial part of the pandemic due to unpredictable disease course. According to international studies, an estimated 7% of children with SARS-CoV-2 are hospitalized, and 28-33% of hospitalized children require intensive care [21].

In our experience, around 58% of patients did not require any respiratory support, 14.74% required oxygen supplementation, non-invasive ventilation (NIV) support was given to 8% of patients, and 16.67% of patients required invasive mechanical ventilation. Another pediatric MIS-C study conducted in the USA [4] also reported a similar figure for invasive ventilation (15%). Initial studies from hospitalized pediatric SARS-CoV-2 patients have reported 5.8% requiring invasive ventilation, 3.9% NIV, and 2.4% high-flow nasal cannula (HFNC); acute respiratory distress syndrome (ARDS) was reported only in 2% [21]. A much higher rate of invasive ventilation was reported in children with acute COVID-19 from PICU from a combined study done in the USA and Canada [15], where 38% of admitted patients required mechanical ventilation.

Average PICU LOS in our data was between seven and 12 days, while average hospital LOS was between 10 and 15 days, varying for different subgroups. In confirmed cases of MIS-C, average PICU LOS as mentioned by Kaushik et al. was 4.7 days with a total hospital stay of 7.8 days [4]. In our study, the stay was significantly higher due to the absence of a step-down facility and greater reliance on PICU for patient care in our hospital. Children fulfilling the MIS-C criteria and those with coexisting illness or requiring respiratory support and/or CRRT were significantly associated with prolonged ICU stay. Our outcome was comparable to other countries in the region with a mortality rate of 4.5% (seven patients), and all the deaths occurred in children with confirmed MIS-C.

Refractory respiratory failure and cardiogenic shock were the most common causes of mortality at our center. Two patients died due to myocarditis-associated cardiogenic shock; one of them also developed acute ischemic stroke leading to brainstem death. Two children died due to septic shock. Of the two patients assigned do not resuscitate (DNR) code status, one died after withdrawal of care as the patient developed signs of brainstem death following severe myocardial depression and hypotensive shock. The other patient was a case of mucopolysaccharidosis and was assigned DNR due to the presence of multiple comorbidities.

A multicentric trial from Saudi Arabia reported a mortality rate of just 0.5%, but the study catered mostly to asymptomatic and less severely affected children (as only one patient required ventilatory support) [13]. Mortality rate for children with MIS-C was nil in a European study by Belhadjer et al. [22] in spite of severe cardiac dysfunction in several of them, as they had good outcomes on extracorporeal membrane oxygenation (ECMO). As many as one-third of those patients had left ventricle (LV) ejection fraction less than 30%, with 80% of the patients requiring inotropic support, 62% were on invasive ventilation, and mechanical circulatory support was needed in 28% of children. This strongly emphasises that even severe cardiac dysfunction in children with MIS-C is transient and with appropriate circulatory support, the outcome is excellent.

A study done exclusively on confirmed cases of MIS-C in the USA by Kaushik et al. [4] reported depressed cardiac function in 65.6% of patients and mortality of 3% as only one child died out of 35 patients, due to hemorrhagic stroke while on ECMO. They reported use of vasopressors in 56% of patients with MIS-C, in contrast to only 26% of patients in our unit. Our hospital did not have facility for ECMO for pediatric patients, and cardiac function could not be assessed in all patients due to various limitations. In our patients, echocardiography was done in only 16% of patients and revealed depressed cardiac function in 8.9% (14 patients), while features suggestive of myocarditis were seen in 3.2% (five patients). Pericardial effusion was seen in 1.9% (three patients). Three patients (1.9%) had some form of congenital heart disease. None of the patients showed features of coronary artery aneurysm.

A retrospective study of SARS-CoV-2-associated deaths in the USA by McCormick et al. (2021) observed that MIS-C contributed to death in just 14%, while the rest of the deaths were attributed to non-MIS-C SARS-CoV-2 [23]. But this study included young people up to 21 years of age, and majority of deaths (58%) occurred in children above 15 years, so the study population was significantly different than that of ours. Some evidence suggests that there is an increased risk of death in children with obesity and chronic neurological disorders [24].

Management

Although the management relied mainly on supportive treatment, a significant proportion of patients required some form of anti-inflammatory as well as anticoagulation therapy. At our center, the most frequently prescribed medication was empirical antibiotics (91.03%), which was quite high and almost double the percentage of that used in a US study [4], but as the frequency of sick children was higher in our PICU, they were given the benefit of the doubt. Steroids were given to 45.51% as administration of steroids was strongly recommended, especially in severely affected population to reduce systemic inflammation. Steroid use was restricted for severe disease and given to patients requiring any form of respiratory support and those fulfilling the MIS-C criteria [25].

Hydroxychloroquine was used in a few patients during the initial stages of the pandemic, and the overall rate of usage was low, as the Food and Drug Administration (FDA) ended the emergency use of hydroxychloroquine and chloroquine for the treatment of COVID-19 in June 2020. The antiviral drug remdesivir was not prescribed in our hospital as it was unavailable, although oseltamivir was given to patients with severe pneumonia, until influenza was ruled out. Several trials have recommended antiviral drugs for adults who require oxygen support as it leads to reduction in the use of mechanical ventilation [26]. However, its use in patients who are already on NIV or mechanical ventilation is not recommended. Recently, there are recommendations for the use of antivirals (remdesivir and ritonavir-boosted nirmatrelvir, Paxlovid (Pfizer, New York, New York, United States)) for non-hospitalized children above 12 years or hospitalized children with or without need for oxygen support (National Institutes of Health (NIH) guideline, based on PINETREE study in adults) [27]. Those children with disease severe enough to require mechanical ventilation or failure of NIV/HFNC should be considered for treatment with tocilizumab. The use of tocilizumab in SARS-CoV-2 infection in children is derived from adult studies (Randomised Evaluation of COVID-19 Therapy (RECOVERY) trial) [28]. Given the already approved use in non-SARS-CoV-2 conditions, its use was started in pediatric SARS-CoV-2-infected patients for immunomodulatory effects. Tocilizumab was used in only three subjects (11.56%) in our study, although data for some of the patients were lost and not included in study. Intravenous immunoglobulin (IVIG) was used in 28.8% (45) patients. The usage was 55.1% (32) in patients with MIS-C, while only 13.2% (13) in the rest of the acute COVID-19 patients. Newer therapies like baricitinib (Janus kinase inhibitor) and tofacitinib were not tried as these medications were introduced during the latter part of the pandemic.

Prophylactic anticoagulation was started with low molecular weight heparin (LMWH, enoxaparin), across all age groups with MIS-C, unless contraindicated. LMWH was used in 32.05% of our patients, and anticoagulation was changed to aspirin in patients needing long-term anticoagulation. A multicentric retrospective cohort study reported thromboembolic complications in 2.1% of SARS-CoV-2-infected children and 6.5% of MIS-C patients [29] for patients above 12 years. No adverse effects with anticoagulation were noted among younger patients (NIH advocates for its use above 12 years).

Among the radiological features, chest X-ray (CXR) precisely, the most frequent findings were infiltrates (33%), followed by cardiomegaly (11%) and lung congestion (5.7%). Features suggestive of ARDS were observed in 3.8%, while pleural effusion was seen in 3.2%. None of the children underwent chest CT scan, and the sick children with respiratory manifestations were monitored clinically and by means of serial X-rays. This was done to avoid unnecessary radiation exposure in the pediatric age group. A case series from a pediatric referral hospital has shown lung consolidation and pleural effusion to be the most frequent CXR findings [30].

Study limitations

One of the major strengths of our study is that it was conducted on a large population of pediatric patients during their hospital admission in a quaternary center in Saudi Arabia which provides preliminary data on the clinical characteristics and outcomes of SARS-CoV-2 infection in children. However, our study does have its share of limitations, such as its retrospective design which could affect the quality of such a pertinent study. Moreover, we could not compose a conclusion on the long-term effects of SARS-CoV-2 infection on children as our follow-up period ended at discharge from the hospital.

Based on the data analysis from this study, we believe further research with a larger sample size from multiple centers and across various regions would yield more information; and follow-up of such a cohort to monitor their long-term outcome is recommended.

Also, the clinical outcomes of other SARS-CoV-2 variants such as Omicron remain to be explored further as it was first reported in Saudi Arabia in December 2021. The COVID-19 vaccine was also introduced for children in Saudi Arabia in December 2021 after our study period ended, so it would be interesting to follow the trends in disease presentation, progression, and outcome through future research as the SARS-CoV-2 virus becomes more endemic or seasonal as compared to the initial pandemic era.

## Conclusions

We have presented our experience with pediatric SARS-CoV-2 infection, which included a large cohort of children from a single quaternary care center. As seen, children of all ages were found to be susceptible. Fever, cough, vomiting, fatigue, and diarrhea were the most common presenting symptoms. Inflammatory markers such as ESR, CRP, serum ferritin, and LDH levels were found to be elevated in nearly all patients. Raised serum lactate and serum creatinine and lymphopenia were of significant note in patients with MIS-C.

The disease presents with a range of severity among infected children; however, most patients responded well to appropriate supportive treatment. A high index of suspicion should be maintained for the possibility of developing MIS-C, necessitating extensive lab and imaging work-up along with frequent clinical assessment. Cardiac assessment is recommended in all patients presenting with severe SARS-CoV-2 infection. Chest CT scan does not seem to add much to the management in the pediatric population, in our opinion. Patients with MIS-C and those requiring respiratory support demonstrated higher mortality rates; additionally, the presence of comorbidities and the need for CRRT were all associated with prolonged length of stay in the PICU.
